# Natural Blockers of PD-1/PD-L1 Interaction for the Immunotherapy of Triple-Negative Breast Cancer-Brain Metastasis

**DOI:** 10.3390/cancers14246258

**Published:** 2022-12-19

**Authors:** Maryam Nakhjavani, Sarah Shigdar

**Affiliations:** 1 School of Medicine, Deakin University, Geelong, VIC 3220, Australia; 2 Institute for Mental and Physical Health and Clinical Translation, School of Medicine, Deakin University, Geelong, VIC 3220, Australia

**Keywords:** triple-negative breast cancer, brain metastasis, immunotherapy, natural medicine, blood–brain barrier

## Abstract

**Simple Summary:**

Aggressive types of breast cancer spread to the brain and can form a new tumor there. The treatment of such a tumor is more difficult because there is a membrane around the brain that limits the entrance of drugs. Currently, chemotherapy is the most well-known treatment for these patients, but it cannot pass through that membrane and such patients often die within two years. Here, we looked at some of the drug candidates that are extracted from plants and traditional herbal medicines and these candidates can activate the immune system to kill cancer. We reviewed whether these molecules could pass the brain membrane to activate the immune system inside the brain to kill cancer there.

**Abstract:**

The limited treatment options for triple-negative breast cancer with brain metastasis (TNBC-BM) have left the door of further drug development for these patients wide open. Although immunotherapy via monoclonal antibodies has shown some promising results in several cancers including TNBC, it cannot be considered the most effective treatment for brain metastasis. This is due to the protective role of the blood–brain barrier (BBB) which limits the entrance of most drugs, especially the bulky ones such as antibodies, to the brain. For a drug to traverse the BBB via passive diffusion, various physicochemical properties should be considered. Since natural medicine has been a key inspiration for the development of the majority of current medicines, in this paper, we review several naturally-derived molecules which have the potential for immunotherapy via blocking the interaction of programmed cell death protein-1 (PD-1) and its ligand, PD-L1. The mechanism of action, physicochemical properties and pharmacokinetics of these molecules and their theoretical potential to be used for the treatment of TNBC-BM are discussed.

## 1. Introduction

Triple-negative breast cancer (TNBC) mainly occurs in younger, premenopausal women with a more aggressive nature, i.e., it is more likely to metastasize to other organs such as the brain. Metastatic TNBC (mTNBC) has a poor overall survival of about 17.5 months, which shows its poor prognosis [1]. This is partly due to the fact that TNBC does not overexpress the common targets of breast cancer treatment including estrogen receptors, progesterone receptors, or human epidermal growth factor receptor 2 (HER-2); therefore, the common breast cancer-targeted therapies cannot be utilized in these patients. The mainstay of TNBC treatment has remained chemotherapy for decades, which, due to its non-selective nature, causes adverse reactions and toxicities in patients, leading to decreased patient compliance and increasing tumor resistance to treatment [2]. Consequently, the effort to find novel, targeted therapies for TNBC has been of special interest and attention. As an example, patients with BReast CAncer gene 1/2 (BRCA1/2) mutations undergo treatment with inhibitors of poly(ADP-ribose) polymerase (PARP) [3]. With the hope of boosting the patient’s immune system to kill cancer, one strategy has been to use immunotherapy targeting PD-1/PD-L1, cytotoxic T lymphocyte-associated antigen-4 (CTLA-4), lymphocyte-activation gene 3 (LAG-3), T cell immunoglobulin and mucin-domain containing-3 (TIM-3) and the hedgehog (Hh) and neuropilin-2 (NRP-2) signaling pathway (reviewed in [2]). So far, the most studied immunotherapy target in TNBC belongs to a group of antibodies targeting PD-1 and PD-L1 [4]. A subgroup of TNBC patients overexpressing PD-L1 were studied in the IMpassion130 Trial. The mTNBC patients received a combination of anti-PD-L1 atezolizumab and nab-paclitaxel, which improved their progression-free survival [5]. This led to the US food and drug administration (FDA) approval of atezolizumab for unresectable advanced PD-L1-positive TNBC patients [6]. The mechanism of this drug relies on the interaction of T cells and cancer cells. Following the interaction of the T cell receptor (TCR) and the cancer antigen, PD-1 expressed on cytotoxic T cells (CTLs) interacts with PD-L1 expressed on cancer cells, leading to the inhibition of the activation of CTLs and an “immune escape”. PD-L1 also plays some roles in the proliferation of cancer cells by affecting the mitogen-activated protein kinase (MAPK) pathway [7]. Therefore, blocking PD-L1 has at least two different outputs: Activating the immune system to kill cancer and inhibiting the proliferation of cancer cells [4,7].

TNBC with brain metastasis (TNBC-BM) is the most severe form of mTNBC to treat. This is because the BBB, a complex structure surrounding the brain, is a highly specialized and selective structure that tightly controls and regulates the delivery of necessary materials to the brain to maintain brain homeostasis [8,9]. Several cellular layers that comprise the BBB include the endothelial cells (ECs), pericytes, astrocytes and the basement membrane. The space between the BBB endothelial cells is sealed with more tight junctions compared to endothelial cells in other parts of the body, which makes them impermeable to hydrophilic molecules [9,10]. Moreover, the electrical resistance of ~1000–2000 ohm cm^2^ in the BBB restricts the movement of ionic molecules [11]. Chemotherapeutics have bulky structures with limited access to the brain and the same applies to monoclonal antibodies targeting PD-1 or PD-L1. In addition, these molecules have animal-derived domains and, therefore, naturally inherit a structure that might cause immunogenic reactions. This has made the medicinal intervention of brain tumors challenging, leading to the failure of these options and keeping the overall survival of these patients to less than two years [12].

Natural molecules have always been used as a template for the development of the majority of medicinal treatments. Moreover, research in this area has been ongoing to develop novel treatments for TNBC and mTNBC from natural origins such as ginsenosides, bacopasides and silibinin via inhibiting cell proliferation, angiogenesis, and cell migration mechanisms [13,14,15,16]. Some of these molecules, such as ginsenoside Rk1 and silibinin, have shown an immunotherapeutic potential [17,18]. Furthermore, silibinin crosses the BBB [19] and impairs the activation of signal transducer and activator of transcription 3 (STAT3), which plays roles in the formation of breast cancer brain metastasis [18,20]. Likewise, several herbal molecules have shown potency in inhibiting the interaction of PD-1/PD-L1; however, their potential to traverse the BBB has not been studied. This paper aims at reviewing these molecules and evaluating their potential as immunotherapeutic agents for the treatment of TNBC-BM.

## 2. Molecules

**Figure 1:**
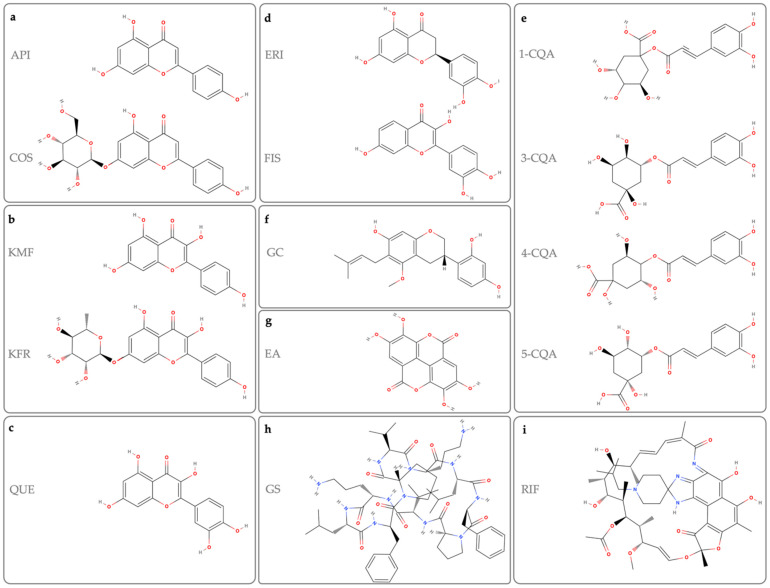
Chemical structure of: (**a**) apigenin (API) and cosmosiin (COS); (**b**) kaempferol (KMF) and kaempferol 7-O-rhamnoside (KFR); (**c**) quercetin (QUE); (**d**) eriodictyol (ERI) and fisetin (FIS); (**e**) 1-caffeoylquinic acid (1-CQA), 3-caffeoylquinic acid (3-CQA), 4-caffeoylquinic acid (4-CQA), and 5-caffeoylquinic acid (5-CQA); (**f**) glyasperin C (GC); (**g**) ellagic acid (EA); (**h**) gramicidin S (GS); and (**i**) rifabutin (RIF).

The majority of the molecules discussed in this paper are flavonoids, which are polyphenolic structures usually found as secondary metabolites in plants. In addition, other natural molecules with heterocyclic and macrocyclic structures are also discussed (Figure 1). Here, we briefly introduce each molecule and then focus on the studies that evaluated the potential of these molecules as blockers of PD-1/PD-L1 interaction.

### 2.1. Apigenin (API) and Cosmosiin (COS)

Apigenin (API) and Cosmosiin (COS) are extracted from the traditional medicinal plant *Salvia plebeia R. Br* (SP) and API and COS share similar structures (Figure 1a). API is a trihydroxyflavone, and COS is API 7-O-beta-D-glycoside, which also exists in an L-glycoside enantiomer form. API, together with some other flavonoids such as quercetin (QUE) and kaempferol (KMF), is the most ubiquitous plant flavonoid among more than 5000 [21]. It has a low toxicity in normal cells compared to cancer cells with antioxidant, and anti-inflammatory properties and influences the induction of apoptosis and cell cycle arrest in cancer cells. These functions are via its effect on several cellular signaling pathways such as PI3K/AKT, MAPK/ERK, and NF-κB Signaling, the Wnt/β-Catenin pathway, STAT 3 and epidermal growth factor receptor (EGFR) (reviewed in [22]). Moreover, studies have shown the efficacy of this molecule in several cancers such as breast, lung and melanoma models [22].

In a study by Choi et al. (2020), the extract of *Salvia plebeia* R. Br. (SPE) blocked the binding of PD-1/PD-L1 in an enzyme-linked immunoassay (ELISA). This was dose-dependent and with specific blocking with no effect on the CTLA-4/CD80 interaction; however, the potency of the SPE was less than a PD-L1-blocking antibody. The blocking effect was attributed to the ethyl acetate fraction of the extract. This fraction of the extract had about eighteen-fold higher amounts of API and eight-fold COS. At 50 mg/mL, the SPE and the ethyl acetate fraction showed an ~42% and ~63% inhibitory action on the PD-1/PD-L1 interaction. At concentrations <50 mg/mL (24 h), the SPE showed no cytotoxicity in a co-culture system containing Jurkat and aAPC/CHO-K1 cells (CHO cells engineered to express a hPD-L1 and TCR agonist). The SPE and the ethyl acetate fraction were used in a co-culture system of aAPC/CHO-K1 cells and Jurkat cells. In this system, a half-effective concentration (EC_50_) of the PD-L1 blocking antibody was ~0.3 µg/mL in activating TCR signaling. The relevant EC_50_ value for the SPE and the ethyl acetate fraction was ~27 and 1 µg/mL, respectively [23]. This demonstrated the importance of the ethyl acetate fraction.

When humanized PD-L1-expressing MC38 cells (hPDL1-MCs) co-cultured with humanized PD-1 mouse splenocytes were exposed with the non-cytotoxic concentrations of SPE, the cell viability was significantly decreased. A co-culture of hPDL1-MCs with CTLs isolated from the tumor showed that cell death was induced by the activation of T cells; however, these effects were not compared with a blocking antibody control. In an hPD-L1 knock-in MC38 tumor-bearing humanized PD-1 mouse model, mice received 5 mg/kg of anti-hPD-1 antibody (intraperitoneal (IP)—twice a week) as the control or 100 and 300 mg/kg of oral SPE. The 100 and 300 mg/kg of SPE inhibited tumor growth by ~45% and 78%, respectively, while the efficacy of the control antibody was 88%. This treatment increased the number of CTLs and CD3+ tumor-infiltrating lymphocytes [23].

Among the seven components of SPE tested at 2 µM as single agents, API and COS showed the best improvement of T cell function, by about two-fold, and also showed a dose-dependent increased T cell function. Both molecules showed a dose-dependent blockage of the PD-1/PD-L1 interaction in an ELISA assay. In both experiments, the COS showed a more effective action. The structure–activity relationship studies confirmed that the monosaccharide group at C_7_ played a major role in the observed effects. This effect of COS was specific to the PD-1/PD-L1 and did not affect the CTLA4/CD80 interaction. The COS showed a dissociation constant (K_D_) of 386 and 85 µM for PD-1 and PD-L1, respectively (R^2^ 0.9804 and 0.9866, respectively). Due to its higher binding rate, the COS had an ~4.5-fold higher affinity for PD-L1 [23]. 

Molecular docking using AutoDock Vina between COS and the crystal structure of hPD-1/hPD-L1 (4ZQK) predicted the binding affinities of −6.2 and −5.8 kCal/mol with PD-L1 and PD-1, respectively. The interaction site was found to have hydrogen bonds between the residues N63, D61, N58 and the glycoside of COS, in addition to a hydrophobic interaction between R131, M115, Q66, and I54 and the backbone of COS (API) [23]. 

### 2.2. Kaempferol and Kaempferol 7-O-Rhamnoside

A variety of edible, non-medicinal and medicinal plants such as *Geranii Herba* produce the flavonol, KMF [24]. KMF is produced to protect plants against oxidative reactions; therefore, an inherent nature of this molecule is its antioxidant property which is important in chemoprevention and anti-inflammatory reactions. Like API, several studies have shown the anticancer properties of KMF in breast, colon and liver cancers [25]. Figure 1b shows the structure of KMF and its glycoside derivative, kaempferol 7-O-rhamnoside (KOR), both of which are found in the extract of *Geranii Herba*. 

In 2020, Kim et al. studied the active ingredients of the *Geranii Herba* extract and showed that it inhibited the interaction of PD-1/PD-L1 (a half inhibitory concentration of (IC_50_) ~88 µg/mL). KMF, among its glycosylated derivatives, was the most potent one (IC_50_~8 µM) with a dose-dependent effect (Table 1); however, its IC_50_ was higher than the controls, neutralizing antibodies and the PD-1/PD-L1 inhibitor C1 [26]. 

KMF or its glycosides showed no cytotoxicity (< 100 µM) on Jurkat and CHO-K1 cells but showed a dose-dependent decreased interaction of PD-1/PD-L1. Both the KMF and KOR showed a similar half-effective concentration (EC_50_) of ~16 µM. The KOR was shown to have a K_D_ of 1.56 × 10^−4^ M. In silico molecular docking studies between KMF and the crystallographic structure of human PD-L1/PD-1 (PDB code: 4ZQK) showed that KMF and KOR attached to PD-L1 at the interaction site of PD-1 with different modes of action (i.e., binding energies of −5.4 and −5.6 kcal/mol, respectively) [26]. It was decided that the glycoside group was associated with the functional activity of KOR in blocking the PD-1/PD-L1 interaction. The binding scores were not compared with a control molecule and, therefore, it cannot be concluded whether this interaction is a strong one or not. 

### 2.3. Quercetin

The other abundantly found flavonoid in fruits and vegetables such as broccoli, onion, pepper and apple is QUE. As shown in Figure 1c, QUE has a similar backbone to KMF, API and their derivatives and, therefore, similar anti-inflammatory and antioxidant actions can be expected. Its pro-apoptotic properties, induction of cell cycle arrest and DNA damage have made QUE a good anticancer candidate [27,28]. Some of the suggested anticancer mechanisms of action of QUE include a decreased production of cyclooxygenase and lipoxygenase and its effect on some signaling pathways such as NF-κB, ERK, and JNK [29,30,31]. In addition, QUE, via inducing the expression of interferon-γ(IFN-γ) and interleukin-4 (IL-4) and promoting the natural killer (NK) cell function, improves the immune system [32,33,34]. Moreover, due to its anti-inflammatory effects, QUE has shown efficacy in several disease models including infection and cardiovascular disease.

Jing et al., in 2021, used an ELISA system on a library of 1018 compounds and showed that 5 µM of QUE-dihydrate showed the best (80%) and dose-dependent inhibition of a PD-1/PD-L1 interaction with an IC_50_ of ~0.2 µM [35]. At 5 µM, the QUE showed a 50% inhibition of the PD-1/PD-L1 interaction. Table 1 summarizes the results obtained on each molecule. 

These results are not comparable to those of KMF, as different techniques were applied to evaluate the inhibitory actions. This study also showed that QUE had a stronger interaction with PD-L1 (K_D_ PD-L1 4.53 µM vs. PD-1 10.19 µM). It was previously shown that the interaction of PD-1/PD-L1 is in the glycosylated form of the PD-L1 [36] and that the QUE inhibited the binding of these glycosylated proteins (IC_50_ 0.5 µM) (Table 1). In a co-culture system of Jurkat and cancer cells (MDA-MB-231 and H460), QUE potentiated the activity of Jurkat T cells causing about a 40% cancer cell death. Furthermore, in a xenograft mouse model, 60 mg/kg of QUE inhibited tumor growth, the population of cytotoxic T cells increased and the expression of cytokines such as interferon-gamma (IFN-γ) and granzyme B in the tumor microenvironment increased to kill the tumor [35]. 

### 2.4. Eriodictyol and Fisetin

*Toxicodendron vernicifluum* (TV) or *Rhus verniciflua* Stokes is another traditional herbal medicine native to China, India, Japan, and Korea and is a source of flavonoids and polyphenols such as eriodictyol (ERI), fisetin (FIS) and QUE [37]. ERI and FIS share a similar backbone structure to those of API, KMF, and QUE (Figure 1d) and have also shown some anticancer potential in several cancer models such as breast, colon, and pancreas cancers [38,39,40,41,42]. In 2020, Li et al. showed that the extract of TV (TVE) inhibited the interaction of PD-1/PD-L1 in a dose-dependent manner (IC_50_~26 µM in a competitive ELISA—Table 1). The efficacy of TVE was attributed to the ethyl acetate fraction. TVE, at 5 µg/mL showed an ~30% inhibitory action on the interaction of CTLA4/CD80, with the ethyl acetate fraction being the most effective [37]. Among several active ingredients in TVE (e.g., ERI, FIS, protocatechuic acid, and caffeic acid), ERI and FIS showed a potent and specific blocking of the PD-L1/PD-1 interaction (IC_50_ 0.04 µM) with no effect on the CTLA4/CD80 interaction [37]. Based on the presented results, this low IC_50_ seemed to be higher than the IC_50_ of the control, i.e., the PD-L1 inhibitor C1 (value not reported in the paper). Additionally, the binding affinity of these molecules needs to be studied.

### 2.5. Caffeoylquinic Acid

Caffeoylquinic acids (CQAs) are a group of phenolic molecules with a quinic acid core that is acetylated with caffeoyl groups (Figure 1e). CQAs have shown a wide range of therapeutic activity such as antioxidant, antibacterial, anticancer, antiviral, and anti-Alzheimer’s activities (reviewed in [43]). In 2018, Han et al. compared the affinity of several mono-CQAs (e.g., 1-CQA, 3-CQA, 4-CQA and 5-CQA) and di-CQAs (e.g., 1,3-diCQA, 1,5-diCQA, 3,4-diCQA, 3,5-diCQA, and 4,5-diCQA) to the affinity of PD-1 and PD-L1. The K_D_ for the PD-1/PD-L1 interaction was 0.17 µM, while the CQAs showed a weaker but comparable affinity of 0.50–0.81 µM. A surface plasmon resonance competition assay showed that the mono-CQAs had a better inhibitory action on the PD-1/PD-L1 compared to di-CQAs. The IC_50_ values of 1-, 3-, 4- and 5-CQA were ~87, 37, 38 and 45 µM, respectively [44].

### 2.6. Glyasperin C

Glyasperin C (GC) is a methoxyisoflavan derivative (Figure 1f) that has been extracted from the ethyl acetate fraction of the traditional herbal medicine, *Glycyrrhiza uralensis*. The bioactive compounds existing in this fraction were determined to be 10 flavonoids, 4 coumarins and 2 benzophenones. At 100 µM, a 30–65% inhibitory action on the PD-1/PD-L1 interaction was observed with these molecules with the GC showing the highest inhibitory action [45]. This makes GC another potential candidate. The backbone structure of GC shares some similarities with the previously mentioned flavonoids (Figure 1) and, therefore, this mechanism of action could be expected.

### 2.7. Ellagic Acid

Ellagic acid (EA) is a chromene-dione derivative that has a hydrophobic moiety of two hydrocarbon rings and a hydrophilic moiety of four hydroxyl groups and two lactones (Figure 1g). It is found in a variety of fruits, vegetables and seeds and has several medicinal activities including anticancer, neuroprotective, anti-inflammatory, antioxidant, hepatoprotective, and skin protection actions (reviewed in [46]). The fruit of *Rubus coreanus* Miquel, commonly known as black raspberry, has been used in traditional herbal medicine for centuries. The extract of the plant (RCE), which contains polyphenolic and flavonoid molecules such as QUE and EA, has antioxidant and anti-inflammatory effects [47,48,49]. 

Kim et al. in 2020, used RCE in a competitive ELISA and showed a dose-dependent inhibition of the PD-1/PD-L1 interaction (IC_50_~84 µg/mL), vs. that of anti-PD-L1 antibody, ~1.7 µg/mL. The RCE was non-cytotoxic on aAPC/CHO-K1 and Jurkat cells at <100 µg/mL. In a co-culture system containing these two cell lines, the RCE activated TCR (EC_50_~56 µg/mL), and at 100 µg/mL it increased the activation of T cells as indicated by an increased production of interleukin 2 (IL-2) by 1.8-fold compared to an untreated control. 

In a humanized PD-1 mouse model, 50 and 100 mg/kg of orally administered RCE decreased the tumor growth rate by 67% and 74%, respectively. The anti-hPD-L1 antibody at 5 mg/kg showed a 95% decreased tumor growth. None of the treatments affected the mice’s body weights [50]. 

EA is the major constituent of RCE. IC_50_ of EA in blocking the PD-1/PD-L1 interaction in a competitive ELISA assay was ~23 µg/mL. A Western blot analysis showed that EA interacted with both PD-1 and PD-L1. Up to 120.9 µg/mL, EA was non-cytotoxic to Jurkat cells and showed a minor decreased viability in aAPC/CHO-K1 cells at 7.56 µg/mL. At a concentration < 7.56 µg/mL, the EA blocked the PD-1/PD-L1 interaction and showed a dose-dependent increase in IL-2 production [50].

### 2.8. Heterocyclic Compounds

Lung et al. (2020) used the ZBC natural product dataset (180,000 molecules) and 5J89, the dimer structure of the PD-L1 IgV domain protein data bank, to perform a virtual molecular docking screening and contact fingerprint analysis. The top 22 selected molecules were subject to in vitro testing using an AlphaLISA PD-1/PD-L1 binding assay and two molecules, i.e., ZINC67902090 ((3S,3aR,6S,6aR)-N6-[4-(3-fluorophenyl)-pyrimidin-2-yl]- N3-(2-pyridylmethyl)-2,3,3a,5,6,6a-hexahydrofu) and ZINC12529904 (1-isopropyl-3-[(3S,5S)-1-methyl-5-[3- (2-naphthyl)-1,2,4-oxadiazol-5-yl]pyrrolidin-3-yl]urea), inhibited the interaction by 30 and 40%, respectively. The ZINC12529904 was more potent than the ZINC67902090 in increasing the PD-L1 dimerization [51]. 

### 2.9. Gramicidin S 

Gramicidin S (GS) is an antibiotic produced by the bacterium, *Bacillus brevis*, which is active against some bacteria and fungi. GS is an amphiphilic molecule with a stable β-sheet with hydrophilic and hydrophobic residues (Figure 1h). Consequently, due to the amphiphilic properties of the interaction surface of PD-L1 with PD-1, Sun et al. used GS as an anti-PD-L1 candidate [52]. The GS showed a weak inhibitory action on the PD-1/PD-L1 interaction (~7%), while a synthesized derivative of GS, namely, Cyclo(-Leu-DTrp-Pro-Thr-Asp-Leu- DPheLys(Dde)-Val-Arg, showed a high potency of 95% at 20 µM and a low IC_50_ of 1.42 µM [52].

In a B16F10 tumor-bearing mouse model, 40 mg/kg of GS (IP) plus anti-CD8 antibody reduced the tumor volume and tumor weight by~55% and 65%, respectively, while this molecule increased the level of CD3+ T cells and CD8+ CTLs [52].

### 2.10. Rifabutin (RIF)

RIF is a macrocyclic antibiotic mostly known as a treatment for tuberculosis (Figure 1i). Using an AlphaLISA human PD1–PDL1 binding assay, Patil et al. (2018) screened RIF together with 19 other FDA-approved macrocyclic molecules for their inhibitory action on the PD-1/PD-L1 interaction. The positive control was an anti-human PD1 antibody with an IC_50_ of 400 ng/mL. In this assay, at 50 µM, rifampin showed an inhibitory action of 48%.

Then, the efficacy of rifampin was compared with four other orally available molecules of this class: RIF, 3-formyl rifamycin, rifamycin SV, and rifapentine. The RIF and rifapentine showed the highest inhibition by ~68% and 52%, respectively. The RIF, rifampin and rifapentine all showed a dose-dependent inhibition of the PD-1/PD-L1 interaction, while the best IC_50_ belonged to the RIF (25 μM). Based on molecular docking studies, RIF formed a stable complex via several hydrogen bonding and π–π interactions [53]. 

## 3. Druggability of the Candidate Molecules to Brain Tumors

The passive diffusion of molecules across a BBB requires a kinetic process with a plasma concentration high enough to produce a sufficient drug concentration at the receptor in the brain [54,55]; however, the concentration is not the sole parameter here. The parameters that affect the passive transport of molecules across the BBB and the PK of the above-mentioned molecules are discussed below.

### 3.1. Physicochemical Properties

The solubility of a drug, which is a function of the physicochemical properties of the molecule, plays a pivotal role in determining the fate and therapeutic efficacy of the drug. For the molecule to be water soluble, H_2_O molecules should break the intermolecular and intramolecular forces; therefore, the water solubility is dependent on the bulk properties of the molecule, and the placement of the polar and non-polar residues and areas [54,55]. Moreover, drug molecules reversibly bind to blood proteins at different levels. It is not yet conclusive whether high protein binding is beneficial towards drug delivery to the brain. For example, albumin or its complexes with drugs cannot traverse the BBB; however, exceptions such as benzodiazepines, steroids or some hormones have high central nervous system (CNS) concentrations rather than their unbound plasma concentrations [56]. The potential explanations include changes in the conformation of the protein in interaction with the capillary walls [57,58,59], protein-mediated transport, especially with AAG [60] and a more permeable structure of the endothelium in some parts of the BBB [61,62].

The brain-to-blood drug concentration ratio (BB) expressed as Log_(BB)_ at a certain time point (Equation 1) has been questioned [63] and the BBB permeability-surface area (PS) or the BBB permeability coefficient, as a quantitative measure of the rate of drug transport (Equation (2)) using in situ vascular perfusion techniques is added as another indicative measurement [56,64,65]:

(1) $${\mathrm{Log}\;}_{\left(\mathrm{BB}\right)}=\frac{Drug\;concentration\;in\;brain}{Drug\;concentration\;in\;plasma}$$

(2) Log (PS)=Observed permeability across BBB (cms)Surface area of brain capillary endothelium (cm2g)

Some of the factors affecting the uptake of a drug from the blood into any given tissue include the blood flow to the tissue, the permeability of the endothelial cells, and the amount of drug available for uptake. The brain tissue is highly perforated however, and the microvascular wall is not permeable to most drugs. The amount of a drug is inversely related to the area under the plasma concentration-time curve (AUC) which is an indication of systemic clearance [64].

The lipophilicity of a molecule is determined using the partition coefficient (LogP) between oil (octanol) and water and is one of the important determinants in drug discovery. High LogP values show low water solubility and poor absorption and usually lead to rapid and high metabolism. This also increases the chance of non-specific binding to hydrophobic molecules and, therefore, a related toxicity [55]. Based on initial studies by Hansch et al., an optimal LogP = 2 showed the highest biological activity in barbiturates [66]. It has also been demonstrated that the optimal LogP for a BBB penetration is 1.5–2.7 with 0 < LogD < 3, and Clog 2.5 [66,67,68,69].

Molecular weight (MW) also plays an important role in the delivery of a drug across the BBB. Regardless of the lipophilicity, a 400 Da cut-off was considered for the MW of drug candidates [70]. Meanwhile, it was also shown that candidates can be divided into three groups based on the relationship between the PS and LogP/MW^2^. Those molecules that have a good correlation or have a greater PS value than their LogP can use passive diffusion and facilitated transport mechanisms. For those with a smaller PS value than their LogP, the MW of a molecule is greater than 400 Da [64]. Marketed CNS drugs, for example, have a mean MW value of 310 [71].

Many other QSAR factors are also important to consider. Hydrogen bonding is a fundamental QSAR factor and is related to the count of heteroatoms, hydrogen bond donor and acceptor counts, polarity, and the polar surface area (PSA). The sum of oxygen and nitrogen counts (O + N), which measures the hydrogen bond acceptors, when less than 5, meets the requirement for CNS penetration [72]. A higher hydrogen bond potential decreases the penetration into the BBB. The average O + N for marketed CNS drugs is 4.32, with hydrogen bond acceptors and donors of 2.12 and 1.5, respectively, and an average %PSA (polar surface area/total surface area ×100) of 16.3% [71]. CNS drugs have a generally lower PSA than other drugs, being about 60 to 90 Å^2^ [68,73]. The rotatable bond counts and the number of rings that account for a molecule’s conformations affect a molecule’s volume. For orally administered drugs, a rotatable bond count > 10 is now correlated with a decreased bioavailability [74], while this count is usually <5 for CNS drugs [71]. Strong acids and bases cannot penetrate the BBB. Meanwhile, the penetration of molecules to lipids is a function of the lipophilicity of the molecules and the concentration of their neutral species. CNS drugs are mostly basic and, therefore, in physiologic conditions, they are charged. At pH 7–8, having a positive charge or tertiary nitrogen is in favor of permeation to the BBB [75,76]. Moreover, a pKa limit of 4–10 has been considered for the penetration of drugs into the BBB [77]. Table 2 summarizes the physicochemical properties of the candidate herbal-derived molecules. The table shows that these molecules do not meet the required conditions described here to penetrate the BBB. 

### 3.2. Pharmacokinetic (PK) Properties

In addition to the physicochemical properties, the PK properties of a molecule are also a determinant of its druggability. For orally administered drugs, the first-pass metabolism effect (FPE), occurring especially in the liver and intestines, has a major effect on the bioavailability of drugs. A rapid FPE decreases the required systemic level of a drug and increases its elimination. In an ideal case, 60 min after the administration of a drug, 80% of it should be available in the body [78]. Metabolism occurs via cytochrome p450 oxidation (CYPs) or conjugation. CYPs are responsible for the majority of metabolism. Successful orally administered CNS drugs, for example, should not have a significant metabolism via CYP2D6 or CYP3A4 to avoid any considerable interaction with co-administered drugs [55]. Serum albumin and α_1_-acid glycoprotein (AGP) are two major plasma proteins responsible for drug-protein binding and binding to weak basic CNS drugs (discussed above). For CNS drugs, a low binding affinity (K_D_ < 10 µM) to albumin is suggested [55]. Here we have a look at the PK parameters of the candidate molecules. The PK factors that help with the comparison of these candidate molecules include the highest plasma drug concentration (C_max_), the time to peak drug concentration (T_max_), the AUC from time 0 to the last measurable concentration (AUC_(0-t)_) and the half-life (T_1/2_)

The PK of API was evaluated in a few studies. An oral administration of 13.5 mg/kg of API to rats had an approximate C_max_ of 42 ng/mL, T_max_ 0.5 h, AUC(0-t) 659 ng×h/mL and T_1/2_ 2 h [79]. Increasing the dose to 60 mg/kg increased these values to an approximate C_max_ 1330 ng/mL, T_max_ 2.5 h, AUC(0-t) 11,763 ng×h/mL and T_1/2_ 4.2 h [80]. The intravenous (IV) administration of 20 mg/kg of API to rats showed a C_max_~11,000 ng/mL, AUC(0-t) ~3300 ng×h/mL and T_1/2_ 1.75 h [81]. The relative bioavailability of API is about 30%, which is considered to be low for human consumption, where a minimum of 50% is needed [82]. The poor bioavailability of API is partly due to FPE, and enterohepatic/enteric recycling which delays its elimination [83]. In comparison, IV administration of 18 mg/kg of COS to rats showed a much lower C_max_ of 0.68 ng/mL, AUC(0-t) 1.34 ng×h/mL and T_1/2_ 2.03 h. This shows that API has a better PK profile compared to COS. 

API has a large volume of distribution (Vd) greater than the total body water of rats (0.67 L/kg). For example, a Vd of ~16 L/kg after a 20 mg/kg IV dose [81] or 2 L/kg after a 5.4 mg/kg dose [84]. This shows the distribution and tissue accumulation of API. In silico studies suggest that API binds to human serum transferrin glycoprotein [85]. The LogP 2.7 makes API a lipophilic agent that should be able to penetrate the cell membrane and BBB [86] and due to a small MW, it can interact with several cell components [87].

Data on KMF is not as conclusive as API. An IV administration of 1, 2 and 4 mg/kg of KMF to rats showed a rapid clearance (4.40–6.44 L/h/kg), while its bioavailability after a 5, 10, and 20 mg/kg oral administration was poor due to an extensive metabolism [88]. Higher doses of 10, 25 mg/kg IV and 100, 250 mg/kg oral were also tested in rats, which also confirmed a high clearance rate of the molecule (3 L/h/kg), a large Vd of 8–12 L/kg and a terminal T_1/2_ of 3–4 h. The oral administration showed a rapid absorption (T_max_~1–2 h), though the bioavailability was still poor (2%). This low bioavailability was attributed to an extensive gastrointestinal and liver metabolism [89]. An administration of 10 mg/kg of KMF to rats showed that the metabolism of KMF is mostly via Phase II metabolism, which concerts KMF to metabolites such as KOR, KMF-7-sulphate and KMF-3-glucuronide, with the latter being the major one. More importantly, the expression of efflux transporters such as BCRP, MRP-1 and -2 also affect the level of KMF conjugates [90]. KMF is suggested to be a substrate of efflux proteins, which can improve the bioavailability of other chemotherapeutics such as etoposide and QUE [91,92].

In rats, 10 mg/kg of QUE as an IV or in oral doses were administered. The bioavailability of this dose was only 5.3% with about 93% metabolism occurring in the gut and 3% in the liver. The oral dose led to a T_max_ of ~0.08 h, C_max_ of ~0.2 µg/mL, and AUC (0–8) 0.06 h×µg/mL. This study did not suggest any enterohepatic recirculation [93], while the human study, probably due to a higher dose, did suggest an enterohepatic recirculation [94]. An oral administration of 100 mg/kg of QUE in rats showed a T_1/2_ of 0.8 h, T_max_ 0.3 h, C_max_ 842 mg/mL and clearance of 0.8 L/h/kg [95]. This study clearly shows the effect of higher doses on the observed PK parameters. Healthy human cases received 500 mg of QUE, three times a day. This study resulted in an oral clearance of 3.5 × 10^4^ L/h, C_max_~15 ng/mL, T_max_ 3 h, AUC~62 ng/mL h, and a terminal T_1/2_ of 3.5 h [94]. In cancer patients, an IV injection of 60–2000 mg/m^2^ of QUE showed a safe dose of 945 mg/m^2^ with a T_1/2_ 3.8–86 min, clearance of 0.23–0.84 L/min/m^2^ and Vd 3.7 L/m^2^ [96]. 

ERI administered to rats at 20 mg/kg IV showed that R(+)-ERI reached a higher serum concentration compared to L(-)-ERI. The ERI showed a rapid distribution within 1 h and an elimination up to 72 h. The T_1/2_ of R(+)-ERI and L(-)-ERI were about 4 and 3.6 h, respectively. The glucuronidated ERI metabolites did not indicate an enterohepatic recirculation. Enantiomers of the ERI showed a similar Vd of about 4.8 L/kg, which correlates with the lipophilic nature of ERI [97]. 

An IP administration of 223 mg/kg of FIS to mice showed a C_max_ of 2.5 µg/mL at 15 min and a T_1/2_ ~3 h [98]. A 3 mg/kg IV dose of FIS in rats showed an AUC of ~276 mg/kg, C_max_~74 µg/mL, Vd 935 mL and clearance of 111 mL/min [99].

To evaluate the PK of CQAs, 0.16 g/kg of *Ainsliaea fragrans* extract was orally administered to rats. This was equivalent to 0.828 mg/kg of COA, 3.61 mg/kg of 1,5-diCQA, 8.74 mg/kg of 4,5-diCQA, 17.52 mg/kg of 3,4-diCQA, and 15.81 mg/kg of 3,5-diCQA. The CQA and diCQAs were rapidly absorbed with a T_max_ of 0.22–0.5 h and another peak at 4 h which suggests their enteric/enterohepatic recirculation. The T_1/2_ of these molecules were all below 2 h; however, 1,5-diCQA showed a 5–25 times higher peak concentration compared to the other diCQAs [100]. 

To assess the PK of EA, 0.8 g/kg of the *Punica granatum* extract equivalent to 85.3 mg/kg of EA was orally administered to rats. This led to a C_max_ of ~0.2 µg/mL, Vd 334 L/kg, AUC 840 µg g/mL, and plasma T_1/2α_ and T_1/2β_ of 0.7 and 0.5 h, respectively [101]. Several studies have indicated a poor absorption and rapid distribution of EA, which can limit its availability to the tissues [101,102,103]. This is while the administration of EA as a total extract has a better PK profile rather than EA alone [101].

## 4. Conclusions

Brain metastasis originating from breast cancer constitutes the largest portion of brain metastases after lung. Almost 15% of these cases are originated from TNBC, with 12% from Her-2+ and 3% from luminal breast cancers [104]. There are many potent small molecules that are of clinical interest. For example, tyrosine kinase inhibitors (TKIs) are examples that are mostly administered to Her-2+ patients and TKIs have shown an improved progression-free survival in Her-2+ breast cancer brain metastases [105]. The efficacy of TKIs versus pertuzumab (anti-Her-2 monoclonal antibody) in Her-2+ patients is being studied (NCT04760431). Other examples of such TKIs in clinical trials are pyrotinib (NCT03933982 and NCT04582968), sorafenib (NCT01724606), and lapatinib (NCT00263588). Immunotherapy for breast cancer brain metastasis is also under investigation in many clinical trials using haploidentical hematopoietic stem cells, cytotoxic lymphocytes, a dendritic vaccine, and dendritic cells (NCT01782274 and NCT03638765), durvalumab (anti-PD-1 antibody—NCT04711824), and bintrafusp alfa (targeting PD-1—NCT04789668). Due to an overexpression of PD-L1 in a subpopulation of TNBC patients, immunotherapy has found a unique attention; however, due to the challenging delivery of anti-PD-L1 antibodies to the brain, newer candidates are being investigated. The molecules reviewed in this paper have shown some efficacy as blockers of the PD-1/PD-L1 interaction; however, not all these studies have shown a relatively good preclinical evaluation of the molecules and many of them lack animal trials. Another drawback is that due to using different assays and evaluation techniques, it is not easy to compare the efficacy and potency of these molecules together. Therefore, further studies are encouraged to determine the best potential candidates. Moreover, flavonoids are substrates of ABC transporters [106] and many of them have enterohepatic recirculation. The PK studies demonstrate that these molecules lack an appropriate bioavailability to be considered for oral administration. 

In theory and based on the parameters discussed in this paper, these molecules do not have the proper physicochemical properties to cross the BBB; therefore, some other interventions will be required to deliver these drugs to the brain. These interventions could include the application of nanotechnology methods, using nanocarriers targeted to the brain. Some examples are targeted nanoscale immunoconjugates on polymeric scaffolds bound to antibodies for T-lymphocyte-associated antigen 4 (CTLA-4) or PD-1 [107], or aptamers targeting the transferrin receptor-mediated transcytosis and PDGRβ-mediated transcytosis, which are good examples [108,109,110,111]. These aptamers are used as drug carriers, can traverse the BBB using transcytosis and deliver the payload to intracranial tumors. 

Further comprehensive QSAR studies could facilitate finding the best backbone structure and using it for synthesizing more potent molecules. This would facilitate the application of these drugs as immunotherapeutic agents in other severe cancers with low survival rates such as high-grade sarcomas of the limbs [112]. It is, however, important to remember that immunotherapeutic agents, including the natural molecules, are still required to be used as combination and neo/adjuvant therapies together with chemotherapies, and not as single agents.

## Figures and Tables

**Table 1:** Key data on the activity of the natural blockers of PD-1/PD-L1 interaction.

Candidate	Inhibiting PD-1/PD-L1		Interaction with PD-L1		EC_50_—T Cell Activity	In Vivo Studies	
	Potency	IC_50_	K_D_	Binding Score		Dose	Reduced TG ^1^
SPE ^2^	42% (50 mg/mL)				27 µg/mL	100 mg/mL 300 mg/mL	45% 78%
SPE-EA ^3^	63% (50 mg/mL)				1 µg/mL		
API ^4^							
COS ^5^			85 µM	−6.2 kcal/mol			
KMF ^6^		8 µM		−5.4 kcal/mol	16 µM		
KOR ^7^			156 µM	−5.6 kcal/mol			
QUE ^8^	80% (5 µM)	0.2 µM	4.53 µM			60 mg/mL	
TVE ^9^		26 µM					
ERI ^10^		0.04 µM					
FIS ^11^		0.04 µM					
CQA ^12^			0.17 µM				
GC ^13^	65% (100 µM)						
RCE ^14^		84 µg/mL			56 µg/mL	50 mg/mL 100 mg/mL	67% 74%
EA ^15^		23 µg/mL					
GS-d ^16^	95% (20 µM)	1.42 µM					
Rifabutin		25 µM					

^1^ Tumor growth, ^2^ *Salvia plebeia* extract., ^3^ *Salvia plebeia*-Ethyl acetate fraction, ^4^ Apigenin, ^5^ Cosmosiin, ^6^ Kaempferol, ^7^ Kaempferol 7-O-rhamnoside, ^8^ Quercetin, ^9^ *Toxicodendron vernicifluum* extract, ^10^ Eriodictyol, ^11^ Fisetin, ^12^ Caffeoylquinic acid, ^13^ Glyasperin C, ^14^ *Rubus coreanus* Miquel extract, ^15^ Ellagic Acid, and ^16^ Gramicidin S derivative.

**Table 2:** Physicochemical properties of candidate molecules. Data from: https://foodb.ca/ (accessed on 1 November 2022) based on ChemAxon.

Candidate	Formula	MW ^1^	O+N ^2^	LogP	PSA ^3^	pKa a. ^4^	pKa b. ^5^	Charge ^6^	H acc. ^7^	H don. ^8^	R bond ^9^
API	C_15_H_10_O_5_	270.2369	5	2.71	86.99	6.57	−5.4	−1	5	3	1
COS	C_21_H_20_O_10_	432.3775	10	0.44	166.14	7.3	−3	0	10	6	4
KMF	C_15_H_10_O_6_	286.2363	6	2.56	107.22	6.38	−3.9	−1	6	4	1
KOR	C_21_H_20_O_10_	432.3775	10	1.24	166.14	7.08	−3.6	0	10	6	3
QUE	C_15_H_10_O_7_	302.2357	7	1.48	127.45	6.38	−4	−1	7	5	1
ERI	C_15_H_12_O_6_	288.255	6	2.53	107.22	7.85	−5	0	6	4	1
FIS	C_15_H_10_O_6_	286.2363	6	1.81	107.22	6.32	−3.9	−1	6	4	1
1-CQA	C_16_H_18_O_9_	354.3087	9	−0.4	211	3.22	−3.2	−1	8	6	5
3-CQA	C_16_H_18_O_9_	354.3087	9	−0.27	164.75	3.33	−3.2	−1	8	6	5

^1^ Molecular weight, ^2^ Oxygen plus Nitrogen count, ^3^ Polar surface area (Å^2^), ^4^ Strongest acidic, ^5^ Strongest basic, ^6^ Physiological charge, ^7^ Hydrogen acceptor count, ^8^ Hydrogen donor count, and ^9^ Rotational bond count.
